# In Vivo Models for Hypertrophic Scars—A Systematic Review

**DOI:** 10.3390/medicina58060736

**Published:** 2022-05-30

**Authors:** Stefan Rössler, Sebastian Philipp Nischwitz, Hanna Luze, Judith C. J. Holzer-Geissler, Robert Zrim, Lars-Peter Kamolz

**Affiliations:** 1 Division of Plastic, Aesthetic and Reconstructive Surgery, Department of Surgery, Medical University of Graz, 8036 Graz, Austria; sebastian.nischwitz@medunigraz.at (S.P.N.); hanna.luze@medunigraz.at (H.L.); judith.geissler@medunigraz.at (J.C.J.H.-G.); robert.zrim@medunigraz.at (R.Z.); lars.kamolz@medunigraz.at (L.-P.K.); 2 International University of Monaco, 98000 Monaco-Ville, Monaco; 3 COREMED—Cooperative Centre for Regenerative Medicine, JOANNEUM RESEARCH Forschungsgesellschaft mbH, 8010 Graz, Austria

**Keywords:** hypertrophic scar, animal model, model, 3R, systematic review

## Abstract

*Backgroundand Objectives:* Hypertrophic scars following surgeries or burns present a serious concern for many patients because these scars not only lead to an aesthetical but also to a functional and psychological burden. Treatment of hypertrophic scars is challenging because despite various treatment options, a low level of evidence hinders preference of any specific treatment plan. To properly identify new therapeutic approaches, the use of in vivo models remains indispensable. A gold standard for hypertrophic scars has not been established to date. This review aims at giving a comprehensive overview of the available in vivo models. *Materials and Methods:* PubMed and CINAHL were queried for currently existing models. *Results:* Models with mice, rats, rabbits, pigs, guinea pigs and dogs are used in hypertrophic scar research. Rodent models provide the advantage of ready availability and low costs, but the number of scars per animal is limited due to their relatively small body surface, leading to a high number of test animals which should be avoided according to the 3Rs. Multiple scars per animal can be created in the guinea pig and rabbit ear model; but like other rodent models, these models exhibit low transferability to human conditions. Pig models show a good transferability, but are cost-intensive and require adequate housing facilities. Further, it is not clear if a currently available pig model can deliver clinical and histological features of human hypertrophic scars concurrently. *Conclusions:* None of the analyzed animal models can be clearly recommended as a standard model in hypertrophic scar research because the particular research question must be considered to elect a suitable model.

## 1. Introduction

While superficial injuries of the skin heal without any visual consequences, deeper injuries affecting the dermis lead to the development of a scar. If there is any disturbance to the wound healing process, pathological scars like hypertrophic scars (HTS) and keloids can form [1]. Despite existing preventative options, the incidence of hypertrophic scars after surgeries and burns remains very high. While following surgery, patients develop hypertrophic scars in 40 to 70%, burn patients have been observed to suffer from these scars in up to 94% [2,3]. These reddish scars not only lead to an aesthetical problem, but are also accompanied by pain, itchiness, a psychological burden and contractures, which influence range of motion if they span a joint line [3,4,5]. All of these factors illustrate that these scars need to be treated appropriately. Due to a low level of evidence of various treatment options (surgery, cryotherapy, laser therapy, triamcinolone, verapamil, 5-fluorouracil, interferon, botulinum toxin A), there is no optimal treatment plan so far that can be clearly recommended [1,6]. This is indicative that further studies are necessary to improve our understanding of hypertrophic scar pathophysiology, to identify new treatment options and to improve the evidence of existing treatment plans. For further studies, in vivo and in vitro models may be used while the 3Rs (Replacement, Reduction, Refinement) must always be considered in animal studies to ensure efficient and ethically responsible research [7]. Due to the fact that the process of scarring is influenced by many different factors like various cell types (fibroblasts, myofibroblasts, keratinocytes, macrophages, lymphocytes, mast cells), cytokines (IL-1β, TNF-α), growth factors (VEGF, PDGF, FGF, EGF, CTGF, TGF-β1), miRNA, metalloproteases, innervation and tension, it is difficult to create appropriate in vitro models evaluating the interaction of the above mentioned factors for hypertrophic scar research [2,3,8,9]. Currently available in vitro models are insufficient to comprehensively replicate the complexity of living organisms and skin tissue, and make it impossible to include all aforementioned factors, which are thought to affect the pathophysiology of hypertrophic scars [10]. Therefore, an encompassing replacement of in vivo models is not feasible to date. Regarding the 3Rs, it is important to combine different animal models used in hypertrophic scar research, simply because there is no described gold standard model yet. This complicates planning and comparison of studies regarding HTS and delays knowledge progress and identifying new treatment options. This review aims at delivering an overview of current animal models used in hypertrophic scar research and discusses advantages and disadvantages of the different models regarding implementation of the 3Rs, transferability of results to humans, costs, and availability to aid researchers in identifying the most suitable animal model to answer their specific research question.

## 2. Materials and Methods

**Figure 1:**
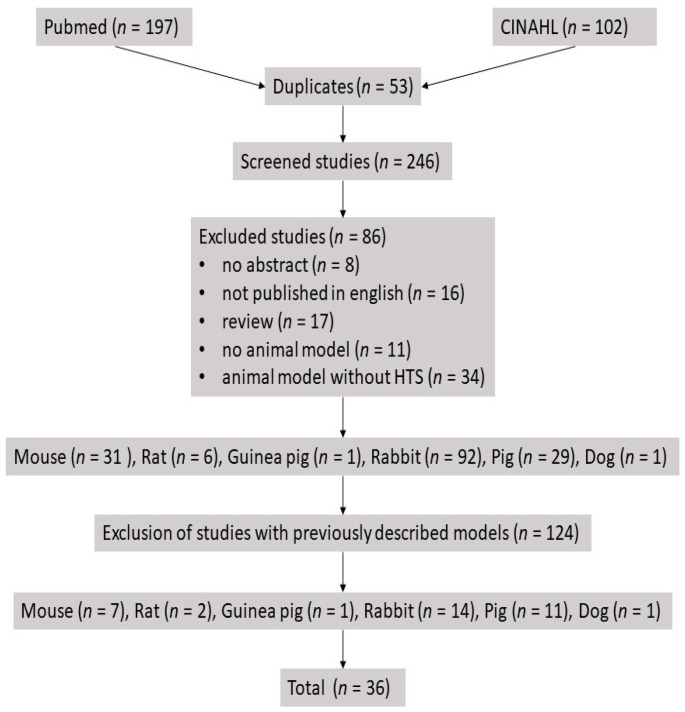
Flowchart. This flowchart shows the literature research procedure. 246 studies were screened, and after determination of exclusion criteria, a total of 36 studies were analyzed.

We clarify that the review was conducted according to the PRISMA guidelines. The PubMed (https://pubmed.ncbi.nlm.nih.gov/ accessed on 22 November 2021) and CINAHL (https://www.ebsco.com/products/research-databases/cinahl-database accessed on 22 November 2021) databases were queried for articles published between 1960 and November 2021 to provide an overview of animal models used in hypertrophic scar research including advantages and disadvantages of each model. PubMed was queried for the MeSH-term “Cicatrix, Hypertrophic AND Models, Animal”, yielding 197 studies; while CINAHL was queried for the term “Hypertrophic scar AND Animal Model“, yielding 102 studies. These studies were transferred to the Mendeley Reference Management Software (Elsevier, Amsterdam, The Netherlands). Subsequently, 53 duplicates were removed using Mendeley, leading to 246 screened studies. The following exclusion criteria were determined: no abstract, not published in English, review, no animal model, animal model without HTS. According to these criteria, 86 articles were excluded. The remaining studies with mouse (*n* = 31), rat (*n* = 6), guinea pig (*n* = 1), rabbit (*n* = 92), pig (*n* = 29) and dog (*n* = 1) models were screened for studies with previously described models without intrinsic novelty. Ultimately, 124 articles were excluded that described no intrinsic novelty in terms of an already used model. The remaining 36 articles with mouse (*n* = 7), rat (*n* = 2), guinea pig (*n* = 1), rabbit (*n* = 14), pig (*n* = 11) and dog (*n* = 1) models were analyzed (Figure 1).

## 3. Results

In this research, hypertrophic scar models with mice, rats, guinea pigs, rabbits, pigs and dogs were identified [5,11,12,13,14,15,16,17,18,19,20,21,22,23,24,25,26,27,28,29,30,31,32,33,34,35,36,37,38,39,40,41,42,43,44,45]. Table 1 summarizes the models and their advantages and disadvantages. Due to the fact that all mentioned animals except pigs have a clearly distinctive fibromuscular layer called the panniculus carnosus beneath their skin, wound healing is dominantly dependent on contracture instead of forming massive amounts of granulation tissue [12,38]. Therefore, specific methods were created to induce hypertrophic scars in these animals as mentioned below.

### 3.1. Mice Models

Regarding mice, three different methods for inducing hypertrophic scars are used. The first method is to transplant human skin grafts onto the back of nude mice, which lack T-cells and have a restricted immune response, and therefore do not reject the skin graft [11,22,41,42]. Using the nude mouse model, hypertrophic scars can be created by burns or incision, while it is also possible to transplant human hypertrophic scar tissue directly onto the animals back [11,41,42]. In the nude mouse model, hypertrophic scars may form without additional wounding, but these are thinner with a lower amount of myofibroblasts, macrophages and mast cells after two months when compared to additional wounded grafts [39]. Hypertrophic scar formation in unwounded grafts is also influenced by the thickness of the skin graft. Wang et al. showed that the use of split-thickness skin grafts leads to relatively thicker scars with a higher number of macrophages, mast cells, fibrocytes and myofibroblasts when compared to the use of full-thickness skin grafts [22]. Hypertrophic scars created in the nude mouse model with transplantation of human full thickness skin grafts without additional wounding form after 20 days and, on average, persist up to 135 days, showing histologic and macroscopic similarities to human hypertrophic scars, while additional wounding of the graft leads to thicker and more contracted scars [39,41]. While the nude mouse model is dependent on the availability of a special mouse strain and human skin grafts, it is also possible to create hypertrophic scars in mice by applying bleomycin or tension [33,40]. Sacak et al. showed that the dermis of mice may begin to demonstrate similarities to human hypertrophic scars after 28 days of continuous bleomycin delivery from an osmotic pump implanted subcutaneously into the back of the animals [33]. Aarabi et al. showed that an increase in mechanical stress by special tension devices during the proliferative phase of wound healing for at least 7 days leads to scars with histopathological similarities to human hypertrophic scars, persisting up to 6 months [40].

### 3.2. The Rat Model

Despite the fact that wound healing in rodents is primarily dependent on contraction instead of granulation tissue formation, wounds in special locations may lead to visible scars [38]. Zhou et al. observed that wounds on the tails of rats heal with more granulation tissue, resulting in reddish scars that were visible for up to 6 months post incision; further, by using customized stainless steel rings with a diameter of 2 cm attached to the rats’ tails, they were able to induce scars with macroscopic, histologic and immunohistochemical similarities to human hypertrophic scars [44].

### 3.3. Rabbit Models

In the rabbit ear model, wounds may be induced by incisions or burns to the ventral side of rabbits ears, resulting in reddish and raised scars at postoperative day 35, while burns lead to 22% larger scars [12,19]. To create up to 12 hypertrophic scars per animal according to this model, wounds must have at least a diameter of 7 mm, and the perichondrium must be removed, which is associated with a delayed re-epithelialization and an enhanced formation of scar tissue [15,38,46]. Removal of the perichondrium may be achieved by conventional surgery, but cryosurgery lowers the risk of damaging underlying cartilage [16]. Zu et al. showed that hypertrophic scarring in rabbits may be induced by subcutaneous injection of anhydrous alcohol into the dorsal trunk [18]. The scars in the alcohol injection model were clearly observable up to 90 days, while scars were starting to flatten 30 days post wounding in the rabbit ear model. Using young rabbits with an age of up to 6 months, gender specific influences on wound healing were insignificant [4]. Further, young animals develop thicker scars than older animals because the proliferation rate of fibroblasts is age-dependent [47].

### 3.4. Pig Models

Besides rodents, pigs also have the potential to develop scars resembling human hypertrophic scars in some aspects [35,36]. In the Duroc pig model, up to 10 hypertrophic-like scars may be induced by incision or burn to the back of the animals, while the depth of the wound correlates with the thickness of the formed scar [27,28,31,32]. Therefore, wounds must have at least a depth of 2.3 mm to re-epithelialize after 30 to 40 days, forming raised and firm scars, distinguishable from surrounding skin with histologic similarities to human hypertrophic scars [27]. These scars demonstrate collagen nodules and numerous myofibroblasts and mast cells compared to uninjured skin, but they do not resemble human hypertrophic scars concerning redness and elevation [31,32]. Further, burn wounds lead to thicker, firmer and more erythematic scars compared to wounds created with a dermatome [36]. Cuttle et al. observed that Large White pigs develop contracted, clearly raised and purple scars with biomolecular similarity to human hypertrophic scars, following wound induction with a remodeled Schott Duran bottle filled with water at a temperature of 92 °C [29].

### 3.5. Miscellaneous Models

Besides mice, rats, rabbits and pigs, it is also possible to induce hypertrophic scars in guinea pigs and dogs [37,45]. Aksoy et al. described hypertrophic scarring in guinea pigs after incision, panniculectomy and coal tar treatment, which lead to the formation of scars with histologic and clinical features of hypertrophic scars like erythema and elevation over the surrounding skin in 50% of the animals; while this method is also accompanied by mortality of 20% due to the toxic effects of coal tar [45]. Kimura et al. showed that full thickness wounds in hairless dogs form hyperpigmented scars rising above unwounded surrounding skin [37]. Compared to haired dogs, these animals form thicker scars with more blood vessels, fibroblasts and inflammatory cells, higher collagen organization and collagen nodules, which is a typical histological feature of hypertrophic scars [37].

## 4. Discussion

To assess the suitability of an animal model, it is important to define the criteria an optimal animal model in hypertrophic scar research should fulfill: In our opinion, an optimal model:

- is ethically justifiable based on the 3Rs;
- forms thick and visible scars with clinical/macroscopic (redness, elevation) and histologic similarities (collagen nodules, mast cells, myofibroblasts) to human hypertrophic scars, and reliably shows no or slow remission over a long period of time;
- exhibits a high transferability of study results onto human conditions, has low costs and is easy availability.

### 4.1. HTS-Models and the 3Rs

As already mentioned, in vivo models cannot be replaced entirely by in vitro models yet, but it is possible to reduce the suffering of animals by electing models that can deliver more than one scar per animal and yield a maximum of new information while relying on a minimum number of animals. Mice or rat models can only have one scar per animal due to their small body surface, while guinea pig, rabbit and pig models may allow more scars per animal, making it possible to dispense with extra control groups, reducing the number of animals required for evaluating new treatment methods. Therefore, multiple-scars-per-animal models like the rabbit ear model, Duroc pig model and Large White pig model are preferable in terms of the 3Rs, while the guinea pig model is questionable due to the tar coal-induced high mortality of the animals, leading to more suffering for animals not contributing to progress in knowledge. Further models with additional devices attached to the animals for a period of time are questionable according to the 3Rs because it is difficult to show that these devices do not lead to strong distress, which must be avoided in ethically responsible research [7]. However, in further studies on the exact mechanisms of tension-related influences on wound healing and hypertrophic scar formation, such models could be useful and might prove indispensable.

### 4.2. Clinical and Histological Similarities to Human HTS

All animals mentioned form scars with histologic and/or clinical criteria of human hypertrophic scars if the right wound depth and size is combined with the right wounding method and, if necessary, additional attachment of special devices is employed. Rodent models form reddish and elevated scars with collagen nodules, which is considered to be a typical criterion of hypertrophic scars, but they lack the same size and thickness as problematic human hypertrophic scars because wound size is limited to body size. This could have an influence on the efficacy of topical treatment methods and impair the transferability of study results onto human conditions. Pig models have the potential to deliver larger scars, but do not fulfill all criteria of human hypertrophic scars. The Duroc pig forms flat, non-reddish scars with collagen nodules, while Large White pigs form reddish and elevated scars similar to human hypertrophic scars [29,32]. However, collagen nodules cannot be detected in the Large White pig model described by Cuttle et al. 99 days after deep dermal thermal injury; they may appear at a later point in time, much like in the Duroc pig model, where collagen nodules cannot be detected in scars less than 5 months old [29,31]. Although collagen nodules are presumed to be a typical histologic feature of hypertrophic scars, Santucci et al. showed that human scars younger than one year do not show nodules, which should be considered when creating further hypertrophic scar models, because the lack of collagen nodules in young scars is not a valid criterion to exclude the existence of a hypertrophic scar [48]. Therefore, further studies using the Large White model are needed, where the histology of the scars after 99 days is examined to establish whether it is a pig model that can replicate scars with clinical and histologic features that resemble human hypertrophic scars.

### 4.3. Differences in Transferability

Another important feature of an optimal animal model is high transferability to human conditions. Pig skin is more similar to human skin than the skin of mice, rats, guinea pigs and rabbits in physiological and anatomical aspects. According to Sullivan et al., human wound healing studies show a concordance of 78% with swine, 53% with small mammals, and 57% with in vitro models [49]. This high concordance makes swine models superior to rodent models concerning the testing of new treatment methods for human use in wound healing and hypertrophic scar research. However, there is a lack of genetically modified swine strains, which makes it difficult to study the influence of various genes on wound healing and hypertrophic scars, while different genetically modified mouse strains are available, allowing researchers to answer such specific questions [50]. Therefore, mouse models play an important role in basic research and identification of new targets for establishing further treatment methods and cannot be banned from wound healing research yet, although study results of swine models show a higher transferability to humans.

### 4.4. Availability and Costs

To ensure that progress in our understanding of hypertrophic scar pathophysiology evolves quickly and that an optimal treatment plan can be established as soon as possible, it is important that the chosen models are readily available and affordable so that research is not limited to institutes that can afford the high cost and provide elaborate infrastructure. Although swine models have the advantage of multiple scars per animal and high transferability, animal welfare in these models is expensive and not every institute possesses adequate housing facilities to rely on swine models in ethically responsible research. Further, at least in our region, the availability of Duroc pigs is restricted because breeders of this particular breed are rare. Large White pigs have a better availability than Duroc pigs but it is unclear if the scars in this model evolve typical histologic criteria of hypertrophic scars. Rodent models with mice, rats, rabbits or guinea pigs are described as easily available and affordable. However, some models require special resources like human skin grafts, tension devices, or bleomycin pumps, which calls the advantage of availability and costs into question. Although exhibiting lower transferability, the rabbit ear model is an interesting alternative to swine models because of high availability, low cost, no need for special devices, and multiple scars per animal. This might be the reason why the number of studies found using rabbit models (*n* = 92) before exclusion of articles with no intrinsic novelty was much higher than for studies using mice (*n* = 31), pigs (*n* = 29), rats (*n* = 6), guinea pigs (*n* = 1), and dogs (*n* = 1).

### 4.5. Does a Universal In Vivo Model for HTS Research Already Exist?

None of the listed models fulfills all of the previously mentioned criteria for an optimal in vivo model, which underscores the importance of special organizations dealing with refinement and reduction of in vivo models [51]. To completely replace in vivo models by in vitro models, further research is necessary. Most of the currently available in vitro models consist of keratinocytes, fibrocytes and dermal matrices [10]. This allows research of epithelial–mesenchymal interactions, but the lack of inflammatory cells impairs research of the inflammatory processes involved in the pathophysiology of hypertrophic scarring. Even if inflammatory cells were added, it would not be possible to research the influence of systemic circulating cells on wound healing and hypertrophic scars. Another alternative to in vivo models in medical research is organ-on-a-chip models. Besides the advantage of research on actual human cells, these models allow permanent perfusion with a nutrient solution, which mimics the in vivo blood supply. Immigration of circulating inflammatory cells into pulmonary and liver tissue has already been discovered [52,53]. Therefore, organ—on-a-chip models may play a role in future hypertrophic scar studies, focusing on the influence of systemic circulating cells or cytokines on hypertrophic scarring and help to reduce animal testing. Another interesting approach to conducting medical research is in silico models. These computer-based models attempt to predict physiological processes and the effects of drugs through special programs and algorithms using data from available studies [54,55]. In silico models could help predict whether a patient is likely to develop hypertrophic scars and requires preventive treatment, or to decide which treatment option is likely to achieve the best results. Koppenol et al. already simulated the influence of myofibroblast apoptosis on dermal thickness and hypertrophic scarring in their in silico model, but simulation of reddish appearance and joint contractures—which are serious problems for patients with hypertrophic scars—was not (yet) possible [56]. To tap into the full potential of in silico models in hypertrophic scar research, further research is necessary. However, these models are dependent on available data sourced from in vivo studies and can therefore not fully replace in vivo models, but they can still reduce medical research’s dependence on them.

## 5. Conclusions

Numerous animal models using mice, rats, guinea pigs, rabbits, pigs and dog models are employed in hypertrophic scar research. None of the existing models can readily be established as a new gold standard model. The 3Rs and model specific advantages and disadvantages must be considered to elect the most suitable model in answering particular research questions. Replacement of in vivo models is desirable, but not entirely feasible quite yet due to a lack of sufficiently complex in vitro and in silico models. Therefore, at least presently, in vivo models are indispensable. If the research question revolves around a general course of scarring or clinical efficacy of therapeutics, we currently advocate using porcine models, while rodents might be better suitable for exploratory basic research questions dealing with specific target sites or pathways. Further studies with the Large White pig model would be interesting because scars in this model show clinical similarities to human hypertrophic scars and may exhibit histologic similarities to human HTS at a later point of time.

## Figures and Tables

**Table 1:** In vivo models in hypertrophic scar research summarizing the advantages and disadvantages thereof.

Model (Animal)	Wound Method	Clinical Features of Human HTS	Histological Features of Human HTS	Multiple Scars per Animal	Transferability to Human Conditions (Animal Specific)	Easy Availability and Low Costs	Critique
Nude mouse model (mouse)	Human skin graft +/− incision or burn	Yes	Yes	No	Low	Yes	Requires human skin grafts, impeded immune system
Bleomycin model (mouse)	Implantation of bleomycin pump	Not clear due to implanted pump	Yes	No	Low	Yes	Requires special Bleomycin pumps, HTS evolves by drug delivery and not by injury
Mechanical stress model (mouse)	Incision + stretching device	Yes	Yes	No	Low	Yes	Requires special stretching devices and continuous control for their adequate positioning
Rat tail model (rat)	Incision + stretching device	Yes	Yes	No	Low	Yes	Requires special stretching device and continuous control
Rabbit ear model (rabbit)	Incision or burn	Yes	Yes	Yes	Low	Yes	Cartilage could influence HTS forming
Duroc model (pig)	Incision or burn	No	Yes	Yes	High	No	Flat scars without redness
Large White model (pig)	Burn	Yes	Not clear	Yes	High	No	No collagen nodules
Coal tar model (guinea pig)	Incision + coal tar treatment	Yes	Yes	Yes	Low	Yes	High mortality
Hairless dog model (dog)	Incision	Yes	Yes	Yes	Unclear	No	Carnivore with fangs and claws

HTS: hypertrophic scars.

## Data Availability

Not applicable.
